# Factors Important to Older Adults Who Disagree With a Deprescribing Recommendation

**DOI:** 10.1001/jamanetworkopen.2023.37281

**Published:** 2023-10-11

**Authors:** Kristie Rebecca Weir, Jenny Shang, Jae Choi, Ruchi Rana, Sarah E. Vordenberg

**Affiliations:** 1 Institute of Primary Health Care (BIHAM), University of Bern, Bern, Switzerland; 2 Sydney School of Public Health, University of Sydney, Sydney, Australia; 3 currently a graduate student at University of Michigan College of Pharmacy, Ann Arbor; 4 Department of Clinical Pharmacy, University of Michigan College of Pharmacy, Ann Arbor

## Abstract

**Importance:**

Little is known about why older adults decline deprescribing recommendations, primarily because interventional studies rarely capture the reasons.

**Objective:**

To examine factors important to older adults who disagree with a deprescribing recommendation given by a primary care physician to a hypothetical patient experiencing polypharmacy.

**Design, Setting, and Participants:**

This online, vignette-based survey study was conducted from December 1, 2020, to March 31, 2021, with participants 65 years or older in the United Kingdom, the US, Australia, and the Netherlands. The primary outcome of the main study was disagreement with a deprescribing recommendation. A content analysis was subsequently conducted of the free-text reasons provided by participants who strongly disagreed or disagreed with deprescribing. Data were analyzed from August 22, 2022, to February 12, 2023.

**Main Outcomes and Measures:**

Attitudes, beliefs, fears, and recommended actions of older adults in response to deprescribing recommendations.

**Results:**

Of the 899 participants included in the analysis, the mean (SD) age was 71.5 (4.9) years; 456 participants (50.7%) were men. Attitudes, beliefs, and fears reported by participants included doubts about deprescribing (361 [40.2%]), valuing medications (139 [15.5%]), and a preference to avoid change (132 [14.7%]). Valuing medications was reported more commonly among participants who strongly disagreed compared with those who disagreed with deprescribing (48 of 205 [23.4%] vs 91 of 694 [13.1%], respectively; *P* < .001) or had personal experience with the same medication class as the vignette compared with no experience (93 of 517 [18.0%] vs 46 of 318 [12.1%], respectively; *P* = .02). Participants shared that improved communication (225 [25.0%]), alternative strategies (138 [15.4%]), and consideration of medication preferences (137 [15.2%]) may increase their agreement with deprescribing. Participants who disagreed compared with those who strongly disagreed were more interested in additional communication (196 [28.2%] vs 29 [14.2%], respectively; *P* < .001), alternative strategies (117 [16.9%] vs 21 [10.2%], respectively; *P* = .02), or consideration of medication preferences (122 [17.6%] vs 15 [7.3%], respectively; *P* < .001).

**Conclusions and Relevance:**

In this survey study, older adults who disagreed with a deprescribing recommendation were more interested in additional communication, alternative strategies, or consideration of medication preferences compared with those who strongly disagreed. These findings suggest that identifying the degree of disagreement with deprescribing could be used to tailor patient-centered communication about deprescribing in older adults.

## Key Points

**Question:**

What are the reasons older adults might disagree with a deprescribing recommendation from a primary care physician in a hypothetical vignette?

**Findings:**

In this survey study, 899 older adults reported valuing their medications, expressed doubts about deprescribing, and preferred to avoid change. Participants who disagreed with the deprescribing recommendation, as opposed to those who strongly disagreed, were more interested in alternative strategies such as improved communication or a replacement medication.

**Meaning:**

These findings suggest that identifying the degree to which older adults disagree with deprescribing recommendations could help tailor patient-centered communication about deprescribing among this population.

## Teaser

This survey study reports the results of a content analysis examining factors that older adults who disagreed with a deprescribing recommendation identify as being important in the decision-making process.

## Introduction

Polypharmacy is a global health issue, with many older adults being prescribed unnecessary or potentially harmful medications.^1,2^ Discontinuation of medications via deprescribing is a safe approach when the harms of medications outweigh the benefits and consideration is given to the individual’s circumstances, level of functioning, goals, and preferences.^3^ The importance of involving older adults in collaborative decision-making about their health is widely acknowledged.^4^ However, deprescribing in clinical practice can be challenging, as older adults are not always supportive of stopping their medications.

While approximately 80% of older adults report being willing to stop a medication if recommended by a health care professional,^5^ 42% to 75% of older adults decline involvement in deprescribing research trials,^6,7,8^ and recommendations for deprescribing have varying rates of acceptance.^9^ However, little is known about why older adults decline deprescribing, as interventional studies rarely capture the reasons. From the qualitative literature, hesitancy toward discontinuing medications has been signaled by older adults and clinicians.^10,11,12,13^ Older adults feel concerned about stopping medications, as communication when starting a medication often emphasizes adherence rather than the anticipated duration of therapy or reasons to stop.^14^

Decision-making about deprescribing among older adults involves several factors. Older adults appear to be more supportive of a deprescribing recommendation that focuses on increased risk of adverse effects of the medication (rationale provided).^15^ Older adults also have different beliefs about a medication to control symptoms right now compared with a medication for preventing a future illness, depending on the specific symptom and future illness in question (medication type).^16,17^ A direct recommendation from a physician to stop a medication is associated with increased experience with deprescribing (physician’s influence).^18^ Finally, older adults have expressed a willingness to deprescribe while also perceiving their medications as beneficial and necessary (beliefs about medications).^5,19^

An initial experimental survey was conducted using hypothetical vignettes to explore older adults’ acceptance of stopping a medication by medication type and the rationale for deprescribing.^20^ Herein, we report the results of a subsequent content analysis among older adults who disagreed with a deprescribing recommendation and examine the factors that participants identified as being important in the decision-making process. To our knowledge, this is the first study to focus on older adults from multiple countries who disagree with a deprescribing recommendation.

## Methods

This survey study was deemed exempt by the University of Michigan Health Sciences and Behavioral Sciences Institutional Review Board from review and informed consent, owing to the use of anonymous data. We followed the American Association for Public Opinion Research (AAPOR) reporting guideline.

### Study Design and Participant Selection

We recruited adults 65 years and older from Australia, the Netherlands, the United Kingdom, and the US to participate in an online study about deprescribing in the context of polypharmacy. Qualtrics randomly routes surveys to eligible panelists who have opted in to receiving online surveys. We requested an equal number of participants per country and 50% female participants. The survey topic was not included in the message to panelists to decrease selection bias.

We asked participants to read a vignette about “Mrs EF,” a 76-year-old woman who routinely takes 11 medications to manage her health conditions (eBox in Supplement 1; the complete vignette is found in Vordenberg et al^20^). Mrs. EF visited her primary care physician (PCP) who recommended that she stop either (1) simvastatin for the primary prevention of heart disease and stroke or (2) lansoprazole to treat indigestion. The rationales given for stopping a medication were either a lack of benefit, potential for harm, or combination of both. Participants were randomized to receive 1 of 6 vignettes.

We asked participants to rate their level of agreement with stopping the medication using a 6-point Likert scale with scale anchors 1 for strongly disagree and 6 for strongly agree. We provided an optional free-text response box and asked participants to tell us why they selected the response. For this content analysis, we subsequently classified participants who selected a score of 1 as strongly disagreeing and a score of 2 or 3 as disagreeing with the deprescribing recommendation. Participants with a score of 4 or higher were excluded from this study.

### Outcomes

The primary outcome was the attitudes, beliefs, fears, and recommended actions of participants in response to the recommendation of deprescribing. The reasons participants agreed and disagreed were substantially different thematically and conceptually; therefore, a content analysis of the responses of participants who agreed (English text only) was conducted separately.^21^

We asked participants to report how many medications they take, their personal experience with the therapeutic class of medication that was presented in the vignette they viewed (hydroxymethyl glutaryl coenzyme A reductase inhibitor [statin] or proton pump inhibitor [PPI]; subsequently referred to as medication experience), and the amount of support needed to manage their medications. We asked participants to self-report their general health and health literacy.^22,23^ Finally, we collected demographic information including age, gender, and educational level. We collected race, ethnicity, nationality, and/or country of origin data based on the standard survey methodology practices in each country. However, we did not have sufficient representation across the 40 response options to draw conclusions about the impact of these factors on disagreement with deprescribing. Therefore, we report data at the level of the country.

### Translation and Coding of Survey Responses

A professional translation service translated the responses from Dutch to English, with 2 translators working on the file to ensure high accuracy. Free-text responses were examined using content analysis, which combines quantitative and qualitative methods to report both the frequency and content of codes.^24^ The comments were organized and coded in Excel, version 2302 (Microsoft Corporation). Four investigators (K.R.W., J.S., J.C., and R.R.) read through all the responses (n = 932) and generated codes that were discussed and modified with all coauthors. The same random set of responses (30 of 932) were coded independently by 2 investigators (K.R.W. and S.E.V.), with high interrater agreement (Cohen κ = 0.8). Discussion between coauthors resolved any remaining conflicts. After coding all 932 responses, 20% were double-coded (S.E.V.) and a Cohen test again indicated high agreement (κ = 0.8).

The full coding framework included 27 codes. One code was used to identify agreement with deprescribing and was used to exclude participants from this study.

### Statistical Analysis

Data were analyzed from August 22, 2022, to February 12, 2023. Descriptive statistics assessed the frequency of each code, and codes with similar meanings were merged. The final analysis framework included 14 themes (fear of worsening symptoms or health, questioning whether the medication is causing problems, concern or fear about the medication being stopped, medication is important or necessary, maintain the status quo or satisfied with existing medication, long-term use of medication, additional information needed prior to deprescribing, second opinion, nonspecific alternative, tests or monitoring, diet or lifestyle change, replacement medication, tapering or reduction, and option to restart) across 6 domains (doubts about deprescribing, values medication, avoidance of change, communication, alternative strategies, and medication preference). We reported the frequency of each theme and domain by participant. We used a χ^2^ test to examine how the information provided in the vignette and older adults’ personal experience and attitudes were associated with the domains and themes. We used a statistical significance level of 2-sided *P* < .05. Analyses were conducted with Stata, version SE 17.0 (StataCorp LLC).

## Results

**Figure 1.**
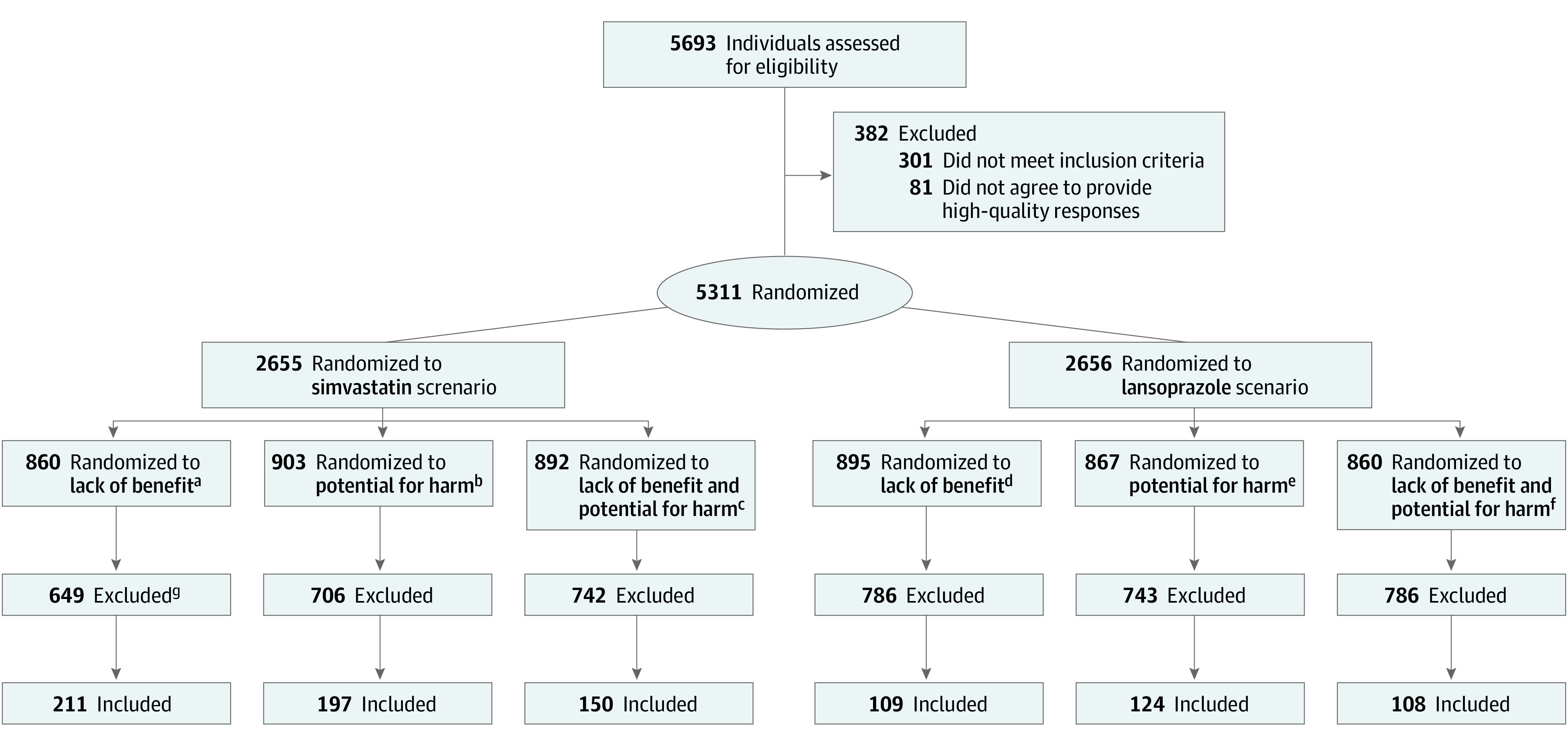
Participant Flow and Study Design ^a^Recommendation: “However, simvastatin may not provide much benefit for people who are your age. Therefore, I recommend that you stop taking simvastatin.” ^b^Recommendation: “However, simvastatin may cause more problems in people who are your age. Therefore, I recommend that you stop taking simvastatin.” ^c^Recommendation: “However, simvastatin may not provide much benefit and it may cause more problems among people who are your age. Therefore, I recommend that you stop taking simvastatin.” ^d^Recommendation: “However, lansoprazole may not provide much benefit for people who take it for more than a few months. Therefore, I recommend that you stop taking lansoprazole.” ^e^Recommendation: “However, lansoprazole may cause problems in people who take the medication for more than a few months. Therefore, I recommend that you stop taking lansoprazole.” ^f^Recommendation: “However, lansoprazole may not provide much benefit and it can cause problems in people who take the medication for more than a few months. Therefore, I recommend that you stop taking lansoprazole.” ^g^Participants who agreed or strongly agreed with deprescribing (n = 4366), provided a free-text rationale without any understandable words (n = 13), or indicated agreement with deprescribing in their free-text rationale (n = 33) were excluded.

A total of 5311 older adults were included in the original study, of whom 932 were included in the coding step of the content analysis (Figure 1). While there was no limit, 95% of free-text responses were allocated up to 3 codes. We excluded 33 responses wherein the participant agreed with deprescribing in free text. The final analytical sample consisted of 899 participants (558 [62.1%] with the simvastatin vignette and 341 [37.9%], the lansoprazole vignette). A total of 494 participants (54.9%) had at least 1 code in the attitudes, beliefs, and fears category, while 413 participants (45.9%) had at least 1 code in the proposed strategies category (eTable in Supplement 1).

### Characteristics of Participants

Participants were a mean (SD) age of 71.5 (4.9) years, 443 (49.3%) were women and 456 (50.7%) were men, and 630 (70.1%) reported obtaining less than a bachelor’s degree (Table 1). Participants most frequently reported being in good health (401 [44.6%]) and were extremely confident filling out medical forms (390 [43.4%]). Participants frequently reported personal medication experience (statin, 345 of 558 [61.8%]; PPI, 172 of 340 [50.6%]).

**Table 1.** Demographic and Medication Characteristics^a^

Characteristic	Respondent group		
	Total (N = 899)	Simvastatin (n = 558)	Lansoprazole (n = 341)
Age, mean (SD), y	71.5 (4.9)	71.6 (0.2)	71.5 (0.3)
Gender			
Male	456 (50.7)	288 (63.2)	168 (36.8)
Female	443 (49.3)	270 (60.9)	173 (39.1)
Country			
Australia	185 (20.6)	113 (61.1)	72 (38.9)
Netherlands	234 (26.0)	132 (56.4)	102 (43.6)
United Kingdom	266 (29.6)	171 (64.3)	95 (35.7)
US	214 (23.8)	142 (66.4)	72 (33.6)
Educational level			
High school diploma or less	303 (33.7)	170 (56.1)	133 (43.9)
Trade school, some college, or associate’s degree	327 (36.4)	212 (64.8)	115 (35.2)
Bachelor’s degree	183 (20.4)	122 (66.7)	61 (33.3)
Master’s degree or higher	86 (9.6)	54 (62.8)	32 (37.2)
Health status			
Excellent	46 (5.1)	30 (65.2)	16 (34.8)
Very good	162 (18.0)	104 (64.2)	58 (35.8)
Good	401 (44.6)	250 (62.3)	151 (37.7)
Fair	245 (27.3)	149 (60.8)	96 (39.2)
Poor	45 (5.0)	25 (55.6)	20 (44.4)
Health literacy (confidence filling out medical forms)^b^			
Extremely	390 (43.4)	242 (62.1)	148 (37.9)
Quite a bit	351 (39.1)	214 (61.0)	137 (39.0)
Somewhat	100 (11.1)	66 (66.0)	34 (34.0)
A little bit	36 (4.0)	25 (69.4)	11 (30.6)
Not at all	21 (2.3)	10 (47.6)	11 (52.4)
Support needed to manage medications^c^			
No support	769 (87.5)	484 (62.9)	285 (37.1)
Occasional support	72 (8.2)	41 (56.9)	31 (43.1)
Complete assistance	38 (4.3)	21 (55.3)	17 (44.7)
Personal use of therapeutic class of medication among participants who received associated vignette			
Never	381 (42.4)	213 (55.9)	168 (44.1)
Currently or in the past	517 (57.6)	345 (66.7)	172 (33.3)
No. of medications used, mean (SD)			
All	7.9 (12.3)	7.3 (0.5)	8.9 (0.8)
Prescription	5.8 (9.9)	5.4 (0.4)	6.5 (0.6)
Over-the-counter and dietary supplements	2.1 (4.3)	2.0 (0.1)	2.3 (0.3)

^a^ Unless otherwise indicated, data in the total column are expressed as No. (%); data in the 2 subset columns, as No. (%) of row total.

^b^ One participant did not answer the question.

^c^ Twenty participants did not answer the question.

### Domains

The attitudes, beliefs, and fears expressed by participants centered around the domains of doubts about deprescribing (361 [40.2%]), values medications (139 [15.5%]), and avoiding change (132 [14.7%]) (Table 2). Participants also identified strategies that may increase their agreement with deprescribing, including improved communication (225 [25.0%]), alternative strategies (138 [15.4%]), and consideration of medication preferences (137 [15.2%]).

**Table 2.** Participant Attitudes, Beliefs, Fears, and Proposed Strategies Reported by Drug, Rationale, and Level of Disagreement

Domain, (No. [%])^b^	Theme	Drug			Rationale				Personal experience with therapeutic class presented in vignette^a^			Level of disagreement		
		No. (%)		*P* value	No. (%)			*P* value	No. (%)		*P* value	No. (%)		*P* value
		Simvastatin (n = 558)	Lansoprazole (n = 341)		Lack of benefit (n = 320)	Potential for harm (n = 321)	Combination of both (n = 258)		Never (n = 381)	Current or past (n = 517)		Strongly disagree (n = 205)	Disagree (n = 694)	
**Attitudes, beliefs, and fears**															
Doubts about deprescribing (361 [40.2])	Fear of worsening symptoms or health	101 (18.1)	64 (18.8)	.80	61 (19.1)	58 (18.1)	46 (17.8)	.92	52 (13.6)	113 (21.9)	.002	47 (22.9)	118 (17.0)	.05
	Questioning whether the medication is causing problems	75 (13.4)	70 (20.5)	.005	33 (10.3)	73 (22.7)	39 (15.1)	<.001	76 (19.9)	69 (13.3)	.008	17 (8.3)	128 (18.4)	<.001
	Concern or fear about the medication being stopped	53 (9.5)	36 (10.6)	.61	32 (10.0)	30 (9.3)	27 (10.5)	.90	48 (12.6)	41 (7.9)	.02	14 (6.8)	75 (10.8)	.09
Values medication (139 [15.5])	Medication is important or necessary	88 (15.8)	51 (15.0)	.74	61 (19.1)	39 (12.1)	39 (15.1)	.05	46 (12.1)	93 (18.0)	.02	48 (23.4)	91 (13.1)	<.001
Avoid change (132 [14.7])	Maintain the status quo or satisfied with existing medication	36 (6.5)	48 (14.1)	<.001	15 (4.7)	28 (8.7)	19 (7.4)	.12	45 (11.8)	39 (7.5)	.03	13 (6.3)	49 (7.1)	.72
	Medication has been used long term	22 (3.9)	40 (11.7)	<.001	37 (11.6)	29 (9.0)	18 (7.0)	.17	36 (9.4)	26 (5.0)	.01	24 (11.7)	60 (8.6)	.19
**Proposed strategies**															
Communication (225 [25.0])	Additional information needed prior to deprescribing	160 (28.7)	65 (19.1)	.001	67 (20.9)	96 (29.9)	62 (24.0)	.03	104 (27.3)	121 (23.4)	.18	29 (14.1)	196 (28.2)	<.001
Alternative strategies (138 [15.4])	Second opinion	37 (6.6)	12 (3.5)	.05	18 (5.6)	16 (5.0)	15 (5.8)	.90	20 (5.2)	29 (5.6)	.81	9 (4.4)	40 (5.8)	.45
	Nonspecific alternative	25 (4.5)	18 (5.3)	.59	12 (3.8)	17 (5.3)	14 (5.4)	.56	19 (5.0)	24 (4.6)	.81	5 (2.4)	38 (5.5)	.07
	Tests or monitoring	26 (4.7)	10 (2.9)	.20	13 (4.1)	13 (4.0)	10 (3.9)	.99	12 (3.1)	24 (4.6)	.26	5 (2.4)	31 (4.5)	.20
	Diet or lifestyle change	13 (2.3)	5 (1.5)	.37	3 (0.9)	7 (2.2)	8 (3.1)	.18	9 (2.4)	9 (1.7)	.51	5 (2.4)	13 (1.9)	.61
Medication preferences (137 [15.2])	Replacement medication	51 (9.1)	27 (7.9)	.53	27 (8.4)	29 (9.0)	22 (8.5)	.96	28 (7.3)	50 (9.7)	.22	11 (5.4)	67 (9.7)	.06
	Taper or reduce	22 (3.9)	31 (9.1)	.001	15 (4.7)	16 (5.0)	22 (8.5)	.10	28 (7.3)	25 (4.8)	.11	5 (2.4)	48 (6.9)	.02
	Option to restart	5 (0.9)	11 (3.2)	.01	6 (1.9)	5 (1.6)	5 (1.9)	.93	5 (1.3)	11 (2.1)	.36	2 (1.0)	14 (2.0)	.32

^a^ Participants who received the simvastatin vignette were asked about their personal experience with hydroxymethyl glutaryl coenzyme A reductase inhibitors while participants who received the lansoprazole vignette were asked about their personal experience with proton pump inhibitors. One participant did not answer the question.

^b^ Represents the number of people who selected at least 1 code in the domain (N = 899).

### Drug

**Figure 2.**
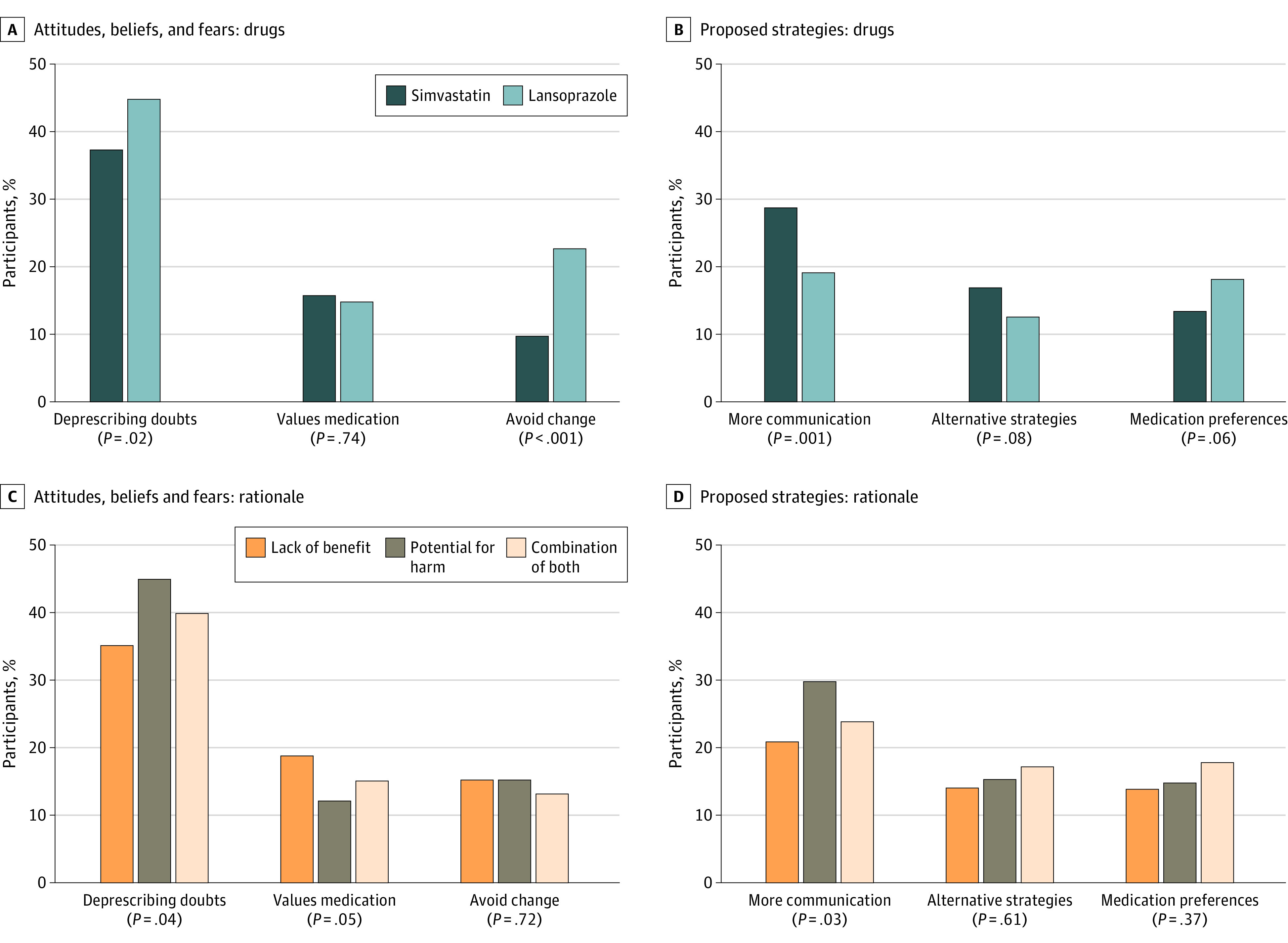
Percentage of Participants With Responses Related to the Information Provided in the Vignette by Domain

Participants who received the simvastatin vignette reported the domain of doubts about deprescribing less often than those who received the lansoprazole vignette (208 [37.3%] vs 153 [44.9%], respectively; *P* = .02) and were less likely to report the theme of questioning whether the medication is causing problems (75 [13.4%] vs 70 [20.5%], respectively; *P* = .01). They were also less likely to report the domain of avoiding change (54 [9.7%] vs 78 [22.9%], respectively; *P* < .001), including the themes of maintaining the status quo (36 [6.5%] vs 48 [14.1%], respectively; *P* < .001) or long-term use of the medication (22 [3.9%] vs 40 [11.7%], respectively; *P* < .001) (Figure 2A).

As it relates to proposed strategies, participants who received the simvastatin vignette more frequently reported the domain of communication, which included the theme that additional information was needed prior to deprescribing, vs those who received the lansoprazole vignette (160 [28.7%] vs 65 [19.1%]; *P* = .001) (Figure 2B). There was no difference in rates of the domain of medication preferences (simvastatin 75 [13.4%] vs lansoprazole 62 [18.2%]; *P* = .06); however, participants who received the simvastatin vignette vs the lansoprazole vignette were less likely to be interested in the themes of tapering or reducing (22 [3.9%] vs 31 [9.1%]; *P* = .001) or the option to restart the medication (5 [0.9%] vs 11 [3.2%]; *P* = .01).

### Rationale

Participants who received the potential for harm rationale (145 [45.2%]) most often reported the domain of doubts about deprescribing compared with participants who received rationales about lack of benefit (113 [35.3%]) and the combination of both rationales (103 [39.9%]; *P* = .04) (Figure 2C). The theme questioning whether the medication is causing problems was most often reported by participants who received the potential for harm rationale (73 [22.7%]) compared with potential lack of benefit (33 [10.3%]) or a combination of both (39 [15.1%]; *P* < .001).

As it relates to proposed strategies, participants who received the potential for harm rationale (96 [29.9%]) most often reported the domain of communication compared with participants who received the rationale of potential lack of benefit (67 [20.9%]) and a combination of both rationales (62 [24.0%]; *P* = .03) (Figure 2D).

### Personal Experience With Medication

**Figure 3.**
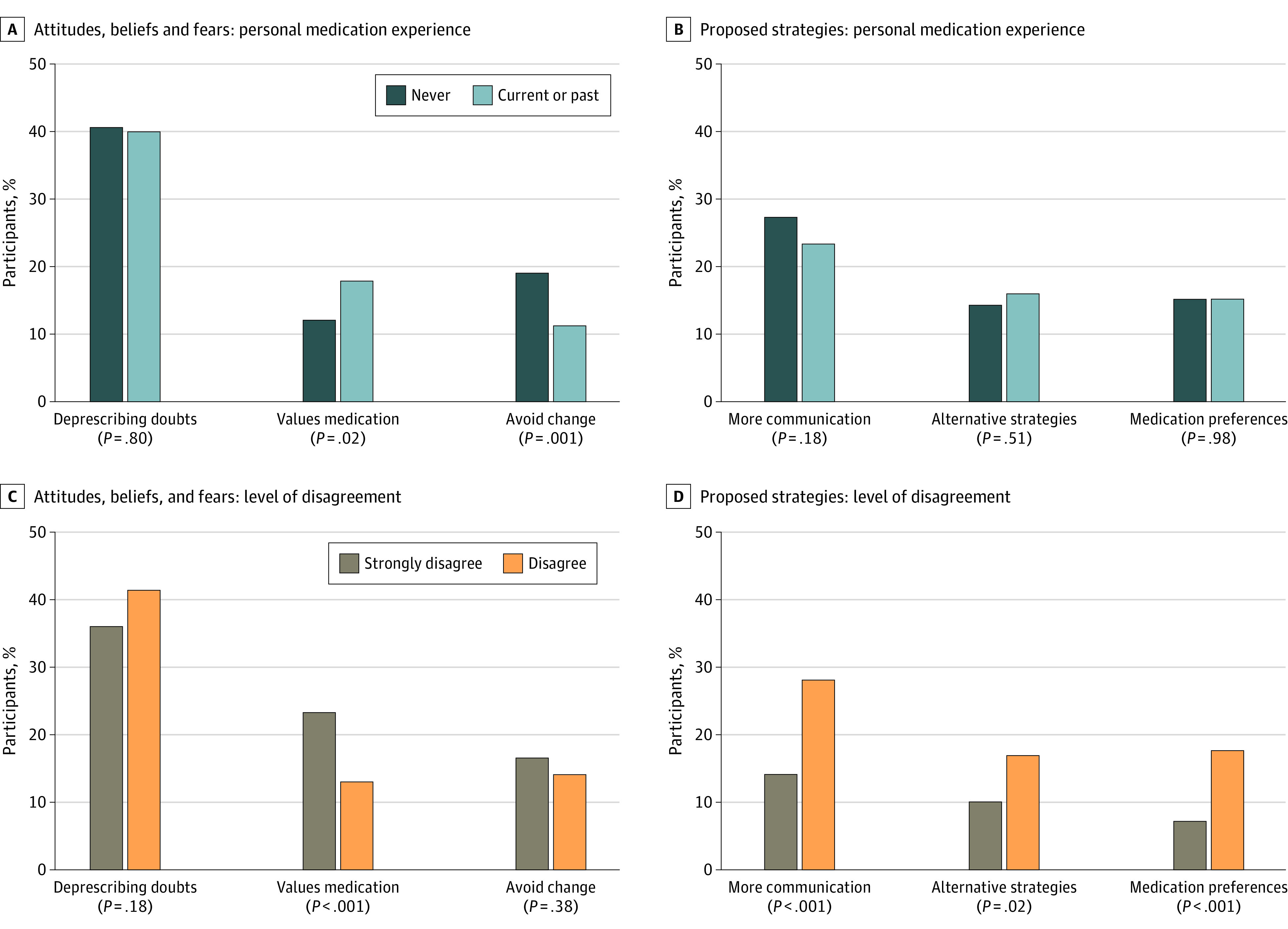
Percentage of Participants With Responses Related to Experience and Attitudes by Domain

Participants with personal medication experience were more likely to report the domain of valuing medications vs those who reported no personal experience (93 of 517 [18.0%] vs 46 of 318 [12.1%]; *P* = .02) (Figure 3A). There was no difference in the overall domain of doubts about deprescribing by personal medication experience (personal experience, 155 [40.7%]; no personal experience, 206 [39.9%]; *P* = .80). However, participants with personal experience were more likely to report the theme of fear of worsening symptoms or health vs with no personal experience (113 [21.9%] vs 52 [13.7%]; *P* = .002) and less likely to report questioning if the medication is causing problems (69 [16.2%] vs 76 [20.0%]; *P* = .008) or concern or fear about the medication being stopped (41 [7.9%] vs 48 [12.6%]; *P* = .02). Participants with personal experience vs no personal experience were less likely to report the domain of avoiding change (59 [11.4%] vs 73 [19.2%]; *P* = .001) or the associated themes of maintaining the status quo (39 [7.5%] vs 45 [11.8%]; *P* = .03) or long-term use of medication (26 [5.0%] vs 36 [9.5%]; *P* = .01). There was no difference in the deprescribing strategies suggested between any domains or themes based on participants’ personal medication experience (Figure 3B).

### Level of Disagreement

Participants who strongly disagreed with the recommendation were more likely to report the domain of valuing medications with the theme medication is important or necessary vs those who disagreed (48 of 205 [23.4%] vs 91 of 694 [13.1%]; *P* < .001) (Figure 3C). There were no differences in the rates of reporting the domain of doubts about deprescribing (strongly disagree, 74 [36.1%]; disagree, 287 [41.4%]; *P* = .18) by level of disagreement. However, participants who strongly disagreed with the recommendation were less likely to report the theme of questioning if the medication is causing problems than those who disagreed (17 of 205 [8.3%] vs 128 of 694 [18.4%], respectively; *P* < .001) (Table 2).

Participants who strongly disagreed were less likely to report the communication domain with the theme of additional information needed prior to deprescribing than those who disagreed with the recommendation (29 [14.2%] vs 196 [28.2%], respectively; *P* < .001) (Figure 3D). Participants who strongly disagreed were less likely to report the domain of alternative strategies than those who disagreed (21 [10.2%] vs 117 [16.9%], respectively; *P* = .02); however, there was no difference in any of the associated themes by level of disagreement. Participants who strongly disagreed vs disagreed were also less likely to report the domain of medication preferences (15 [7.3%] vs 122 [17.6%], respectively; *P* < .001) and the associated theme of tapering or reducing the medication (5 [2.4%] vs 48 [6.9%], respectively; *P* = .02).

## Discussion

This international survey study used hypothetical vignettes to assess older adults’ reasoning for disagreeing with a deprescribing recommendation from a PCP and to explore potential strategies for increasing agreement. Over one-half of participants had personal experience taking a medication in the same therapeutic class as the medication included in the vignette they viewed. To our knowledge, this is the first study to quantify the perspectives of older adults from 4 countries who disagreed with a deprescribing recommendation. Understanding patients’ hesitancy toward deprescribing may help clinicians to better communicate with patients to increase their receptivity to deprescribing recommendations.

There were differences in the rates of the domain of deprescribing doubts for the factors aligning with information provided in the study. Participants who received the simvastatin vignette reported fewer deprescribing doubts but also expressed more interest in additional information via the communication domain compared with participants who received the lansoprazole vignette. Participants who received the rationale stating that continuing the medication could cause harm reported more deprescribing doubts and higher interest in additional communication.

There are several possible reasons why the drug included in the vignette was a factor in older adults’ doubts about deprescribing. In our survey, we included information suggesting that the consequences of inappropriately stopping a statin were more serious (risk of heart disease and stroke) than stopping a PPI (risk of indigestion). There is an ongoing debate regarding the potential benefits and harms of statins for primary prevention in older adults and the risks of deprescribing.^6,25,26,27^ It can be challenging for PCPs to discuss uncertainty in the evidence^28^ while addressing patient fears during a brief patient encounter. Randomized clinical trials of older adults and statin use are ongoing and will provide high-quality, timely evidence; these trials include the Statins in Multimorbid Older Adults Without Cardiovascular Disease (STREAM) trial^29^ in Switzerland and the Statins in Reducing Events in the Elderly (STAREE) trial^30^ in Australia.

Participants who received the lansoprazole vignette were more likely to question whether the medication was causing problems, acknowledge that the medication had been used long term, and preferred to maintain the status quo. These attitudes and beliefs may be more common when patients experience noticeable improvement in their symptoms (eg, indigestion) as opposed to needing laboratory tests to measure effectiveness (eg, lipid panel for statins). Furthermore, participants were more likely to suggest tapering or reducing the dose of lansoprazole, aligning with common practices related to deprescribing PPIs (taper) and statins (abruptly stop). Success rates of deprescribing studies for inappropriate PPIs range from 14% to 64%, and tapering appears to be a more effective strategy than abruptly stopping.^31^ Participants also preferred to have the option to restart the PPI if their symptoms returned. Our findings suggest that recommending a supervised deprescribing trial with the option to restart the PPI (at the same or lower dose) may increase older adults’ agreement with attempting deprescribing.

Studies have found that the potential for harm from a medication affects an individual’s willingness to take it and is a factor in decisions about tradeoffs.^15,32^ In our study, participants were influenced by the rationale for deprescribing provided in the vignette. We indicated the potential for harm and did not directly connect any specific adverse effects that the patient was experiencing with the use of the medication recommended for deprescribing. We did not provide information about what harms might occur or the likelihood of these negative events happening during a specific time. Given that over one-half of participants had personal experience with statins or PPIs, they may have considered their own knowledge and experiences when considering the potential harms. For example, PPIs are regulated as nonprescription products in many countries, and therefore participants may think the harm of using these drugs is low. Similarly, participants may be aware that statins are generally well tolerated, leading them to question whether the survey information was comprehensive. Our research signals the need to explore the combination of the potential for harm rationale—which elicits a strong response—with nuanced information personalized to the patient’s health and social situation.

We found differences in rates of the domain of valuing medications for the factors aligning with the participant’s personal experience. Participants with personal medication experience more frequently reported the domain of valuing medications. Participants who strongly disagreed as opposed to disagreed with the deprescribing recommendation were more likely to report the domain of valuing medications and were less interested in alternative strategies, more communication, or medication preferences. More than one-half of participants reported personal experience with the type of medication in the vignette, which lends credibility to the findings. Participants who have taken these medications likely had a health issue that needed to be addressed (eg, high cholesterol level or indigestion) and they agreed to take a medication. These experiences likely shaped participants’ perceptions about the importance of the medication. Furthermore, qualitative research has found that older adults can be attached to their medicines and can fear the possible negative consequences of stopping a medication over the potential adverse effects of continuing.^19^ This may be magnified by inaccurate perceptions of potential benefits (overestimated) and harms (underestimated) of treatments for both patients and clinicians.^29,30^ When prescribing a new medication, health care professionals should introduce the idea of periodically reevaluating the necessity, effectiveness, and safety of the medication to determine if deprescribing may be appropriate.^31,33^

Over three-quarters of participants reported disagreeing, as opposed to strongly disagreeing, with the deprescribing recommendation. Participants who disagreed were interested in additional communication, alternative strategies, and consideration of their medication preferences than those who strongly disagreed, suggesting that participants who disagree are willing to reconsider the deprescribing recommendation. They may benefit from a detailed discussion of the pros and cons of the options, with time to ask questions and deliberate before making a decision. Conversely, older adults who strongly disagreed with deprescribing were less interested in additional information and alternatives. Therefore, health care professionals should consider not raising the idea of deprescribing in the context of preference-sensitive medication decisions if a patient has previously expressed strong disagreement. However, deprescribing conversations should take place if continuing the medication could cause significant harm. In this context, the key challenge is convincing an older adult that their medication could cause more harm than good, and addressing emotions and fears will be as important as providing information to achieve deprescribing.

### Limitations

This study has some limitations. The survey used hypothetical vignettes that may not reflect the choices participants would make in real life. Participants were not asked about their medical conditions (eg, gastrointestinal tract bleeding) that may have affected their responses. This analysis approach risks oversimplification (eg, rationale for deprescribing) or that contextual factors might be lost. While we sought to standardize coding across team members, we recognize that content analyses include subjective interpretation of the data.

## Conclusions

In this survey study, older adults who disagreed with the deprescribing recommendation were more interested in additional communication, alternative strategies, or consideration of medication preferences compared with those who strongly disagreed. These results suggest that identifying the degree of disagreement with deprescribing could be used to tailor patient-centered communication about deprescribing in older adults.

## Notes

Supplement 1. **eTable.** Prominent Domains and Themes Identified in Free-Text Responses **eBox.** Medical Information for the Hypothetical Patient Vignette

Supplement 2.
Data Sharing Statement
