# Supplementary material for: Factors Important to Older Adults Who Disagree With a Deprescribing Recommendation
Source: JAMA Netw Open. 2023 Oct 11;6(10):e2337281. doi: 10.1001/jamanetworkopen.2023.37281 (PMC10568363; doi:10.1001/jamanetworkopen.2023.37281)
Supplement: Supplement 1. — eTable. Prominent Domains and Themes Identified in Free-Text Responses eBox. Medical Information for the Hypothetical Patient Vignette [file jamanetwopen-e2337281-s001.pdf]

Supplementary Online Content

Weir KR, Shang J, Choi J, Rana R, Vordenberg SE. Factors important to older adults who disagree with a deprescribing recommendation. *JAMA Netw Open*. 2023;6(10):e2337281. doi:10.1001/jamanetworkopen.2023.37281

eTable. Prominent Domains and Themes Identified in Free-Text Responses

eBox. Medical Information for the Hypothetical Patient Vignette

This supplementary material has been provided by the authors to give readers additional information about their work.

© 2023 Weir KR et al. *JAMA Network Open*.

eTable. Prominent Domains and Themes Identified in Free-Text Responses

**Domain**	**Theme**	**Example quote**
Doubts about deprescribing (n=244, 27.1%)	Fear of worsening symptoms or health	*"Because it controls cholesterol and stops your artery's from clogging up and causing a heart attack or a stroke"*
	Questioning if the medication is causing problems	*"She is more in danger of a heart attack or a stroke, the other problems are a 'maybe', the lack of taking the drug is definite"*
	Concern or fear about the medication being stopped	*"As she has been taking this medication for some considerable time to suddenly stop could be more damaging than to continue"*
Values medication (n=139, 15.5%)	Medication is important or necessary	*"It seems to be 1 of the most important medicine"*
Avoid change (n=132, 14.7%)	Maintain the status quo or satisfied with existing medication	*"If she is getting on well with the medication and feels comfortable with it why would she change"*
	Medication has been used long-term	*"She is used to taking it already for years"*
Communication (n=225, 25.0%)	Additional information needed prior to deprescribing	*"More questions need to be asked and answered. What causes the indigestion? What alternatives are there? Why are you suggesting that change?"*
Alternative strategies (n=138, 15.4%)	Second opinion	*"I think I would want a second opinion"*
	Tests or monitoring	*"We do not know what her cholesterol numbers are, so it might depend on that"*
	Diet or lifestyle change	*"Could she try to eat a better fresh food low fat diet and keep the lansoprazole to have only as needed"*
	Non-specific alternative	*"I think she should discuss an alternative or try to solve the indigestion problem another way"*
Medication preferences (n=137, 15.2%)	Replacement medication	*"If [PCP] is going to stop the medication it should be replaced by another drug to help with cholesterol maybe something newer with less side effects"*
	Taper or reduce	*"Should first start with tapering off, then a few weeks without to see what effect it has had. If it goes well then stop completely and if not then just take it again"*
	Option to restart	

© 2023 Weir KR et al. *JAMA Network Open*.

eBox. Medical Information for the Hypothetical Patient Vignette

Mrs. EF has multiple health conditions:	Mrs EF takes 11 medications:
Atrial fibrillation (irregular heartbeat)	
Chronic obstructive pulmonary disease (chronic breathing problem)	Tiotropium
	Albuterol
Constipation	Senna
Depression	Citalopram
High blood pressure	Atenolol
	Hydrochlorothiazide
	Lisinopril
High cholesterol	Simvastatin
History of blood clots	Warfarin
Indigestion (upset stomach)	Lansoprazole
Prevention of brittle bones (osteoporosis)	Calcium

*The full hypothetical patient vignette can be found here in supplementary information: https://doi.org/10.1007/s11606-022-07850-5

Mrs EF, 76 years of age, experiences a number of problems including feeling tired, constipated and occasionally dizzy, and she has told her GP about this previously. It is unclear which, if any, of her medications are causing these problems. (Vordenberg SE, Weir KR, Jansen J, Todd A, Schoenborn N, Scherer AM. Harm and medication-type impact agreement with hypothetical deprescribing recommendations: a vignette-based experiment with older adults across four countries. J Gen Intern Med. 2023;38(6):1439-1448. Medline:36376636 doi:10.1007/s11606-022-7 07850-5)

© 2023 Weir KR et al. *JAMA Network Open*.
