# Supplementary material for: Factors Important to Older Adults Who Disagree With a Deprescribing Recommendation
Source: JAMA Netw Open. 2023 Oct 11;6(10):e2337281. doi: 10.1001/jamanetworkopen.2023.37281 (PMC10568363; doi:10.1001/jamanetworkopen.2023.37281)
Supplement: Supplement 2. — Data Sharing Statement [file jamanetwopen-e2337281-s002.pdf]

Data Sharing Statement

Weir. Factors Important to Older Adults Who Disagree With a Deprescribing Recommendation. *JAMA Netw Open*. Published October 11, 2023. doi:10.1001/jamanetworkopen.2023.37281

Data
Data available: Yes
Data types: Deidentified participant data
How to access data: The data that support the findings of this study are available upon request from the corresponding author. The data are not publicly available due to privacy or ethical restrictions.
When available: With publication

Supporting Documents
Document types: None

Additional Information
Who can access the data: Researchers whose proposed use of the data has been approved
Types of analyses: Specified purpose
Mechanisms of data availability: After approval of a proposal
